# Red Y_2_O_3_:Eu-Based Electroluminescent Device Prepared by Atomic Layer Deposition for Transparent Display Applications

**DOI:** 10.3390/ma14061505

**Published:** 2021-03-19

**Authors:** José Rosa, Mikko J. Heikkilä, Mika Sirkiä, Saoussen Merdes

**Affiliations:** 1 Beneq Oy, Olarinluoma 9, FI-02200 Espoo, Finland; jose.rosa@beneq.com (J.R.); mika.sirkia@beneq.com (M.S.); 2 Department of Chemistry, University of Helsinki, P.O. Box 55, FI-00014 Helsinki, Finland; mikko.j.heikkila@helsinki.fi

**Keywords:** Y_2_O_3_:Eu, phosphor, photoluminescence, electroluminescence, atomic layer deposition

## Abstract

Y_2_O_3:_Eu is a promising red-emitting phosphor owing to its high luminance efficiency, chemical stability, and non-toxicity. Although Y_2_O_3_:Eu thin films can be prepared by various deposition methods, most of them require high processing temperatures in order to obtain a crystalline structure. In this work, we report on the fabrication of red Y_2_O_3_:Eu thin film phosphors and multilayer structure Y_2_O_3_:Eu-based electroluminescent devices by atomic layer deposition at 300 °C. The structural and optical properties of the phosphor films were investigated using X-ray diffraction and photoluminescence measurements, respectively, whereas the performance of the fabricated device was evaluated using electroluminescence measurements. X-ray diffraction measurements show a polycrystalline structure of the films whereas photoluminescence shows emission above 570 nm. Red electroluminescent devices with a luminance up to 40 cd/m^2^ at a driving frequency of 1 kHz and an efficiency of 0.28 Lm/W were achieved.

## 1. Introduction

Inorganic-based electroluminescent (EL) devices have been extensively studied for transparent flat panel display applications due to their distinct characteristics. Such technology allows for the creation of displays capable of withstanding harsh environments thanks to their exclusively solid structure, which leads to a high level of vibration and mechanical shock resistance [1]. Additionally, the electroluminescence phenomenon, which is not affected by temperature, allows EL devices to operate in a wide range of temperatures [2]. Furthermore, the ability to use alternating current to drive EL devices prevents charge accumulation, leading to long operating lifetimes [3]. 

Because the abovementioned characteristics are difficult to achieve with technologies such as organic-light emitting diodes (OLEDs), inorganic-based electroluminescent displays are very attractive from the commercial point of view. LUMINEQ thin film electroluminescent (TFEL) rugged displays and their transparent version TASEL displays are good examples of such commercial products which have been incorporated in industries such as automotive, industrial vehicles, and optical devices.

While yellow and green TFEL and TASEL displays are commercially available, demand for red EL devices has been increasing. Transparent red electroluminescent displays could, for example, be integrated to heavy vehicles, enabling them to display warning signs more effectively, thereby increasing the safety of operators. In the past, some attempts to develop red electroluminescent devices have been made by integrating phosphors such as CaS:Eu [4,5,6], CaY_2_S_4_:Eu [7], β-Ca_3_(PO_4_)_2_:Eu [8], and ZnS:Sm,P [9] into the classic dielectric/semiconductor/dielectric (DSD) EL device structure. Red EL devices, with phosphors such as Eu_2_O_3_ [10], Ga_2_O_3_:Eu [11,12], and IGZO:Eu [13], were also developed using alternative device structures. However, only the use of a color filter with the yellow ZnS:Mn phosphor resulted in sufficiently high red luminescence to be used in commercial products [14]. This solution is unfortunately not suitable for transparent display applications as the use of filters reduces the overall transparency of the device.

Among the currently available red inorganic phosphors, Y_2_O_3_:Eu and Y_2_O_2_S:Eu are the most efficient [15,16]. Y_2_O_3_ and Y_2_O_2_S are known for their good chemical and photochemical stability. Furthermore, because Y^3+^ and Eu^3+^ have similar dimensions of the ionic radii, rare-earth materials such as Eu^3+^ can easily be integrated into Y_2_O_3_ and Y_2_O_2_S matrices [17]. However, Y_2_O_3_ exhibits a high electrical resistivity, with reported values in the 10^11^–10^12^ Ωm range [18], which makes it incompatible with the classic DSD electroluminescent device structure. Nevertheless, several papers have demonstrated the successful use of Y_2_O_3_ and Y_2_O_2_S in red and green electroluminescent devices using multilayer structures where ZnS is used as a carrier accelerating layer [19,20]. 

Y_2_O_3_:Eu thin film phosphors can be grown by various methods such as wet chemistry [21], laser vaporization [22], hydrothermal [23], microwave hydrothermal [24,25], chemical precipitation with calcination [26], co-precipitation [27], Pechini [28], sol–gel [29,30], and pulse laser deposition [31] methods. Atomic layer deposition (ALD) is a well-known method that allows the growth of uniform and dense films with well-controlled stoichiometry and high chemical stability. Moreover, ALD, which is the method used for the fabrication of commercial electroluminescent displays, offers the advantage of an all-in-one growth step for the dielectric and phosphor layers in a DSD structure, thereby improving device resistance to moisture [1,32]. Years of advances in ALD technology have allowed the use of more elements and chemical precursors for the development of novel processes. As a result, opportunities for the fabrication of high-quality phosphors, and consequently more efficient electroluminescent devices, may arise in the future. 

In a previous paper, we reported the growth of blue and red Y_2_O_3-x_S_x_:Eu phosphors by ALD [33]. In this work, we focus on the fabrication and the performance evaluation of Y_2_O_3_:Eu-based multilayer structure electroluminescent devices that can potentially be used in red transparent display applications.

## 2. Materials and Methods

Atomic layer deposition processes for Y_2_O_3_, Eu_2_O_3_, Al_2_O_3_, and ZnS thin films were first developed on (100)-oriented Si substrates. All the films were grown at 300 °C in a Beneq TFS-200 ALD-reactor (Beneq Oy, Espoo, Finland) at a pressure of about 1.3 mbar. (CH_3_C_p_)_3_Y (98%, Intatrade, Anhalt-Bitterfeld Germany), Eu(thd)_3_ (THD = 2,2,6,6-tetramethyl-3,5-heptanedionate) (99.5%, Intatrade, Anhalt-Bitterfeld Germany), Zn(OAc)_2_ (99.9%, Alpha Aesar, Thermo Fisher GmbH, Germany), and trimethylaluminum (TMA, Al(CH3)3) (98%, Strem Chemicals UK Ltd., Cambridge, UK) were used as precursors for yttrium, europium, zinc and aluminum, respectively, while H_2_O and/or O_3_ were used as oxygen precursors for the Y_2_O_3_, Al_2_O_3_, and Eu_2_O_3_ processes. H_2_S was used as sulfur precursor for the ZnS process. In all processes, N_2_ was used as a carrier and purging gas. Details about the pulsing sequences and pulse and purge times are presented in Table 1. The doping level of the Y_2_O_3_ films with Eu was controlled by pulsing M number of Y_2_O_3_ cycles followed by N number of Eu_2_O_3_ cycles, resulting in an M:N doping ratio. To form the Y_2_O_3_:Eu layer, M:N cycles were repeated until achieving the expected thickness, always starting with a Y_2_O_3_ cycle and ending with a Eu_2_O_3_ cycle.

**Figure 1:**
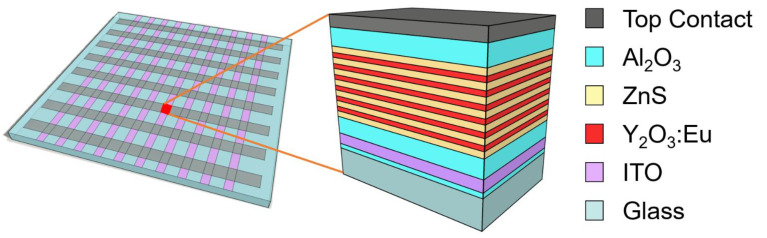
Schematic illustration of the passive matrix-like structure and the device cross section of the Y_2_O_3_:Eu/ZnS EL pixel prepared by ALD. The device is based on the multilayer electroluminescent structure proposed by T. Suyama et al. [19]. In this work, 6 layers of Y_2_O_3_:Eu and 7 layers of ZnS were used.

The electroluminescent device was prepared using the structure proposed by T. Suyama et al. [19]. The multilayer structure was grown by ALD on a standard glass substrate coated with an ion-diffusion barrier and an ITO layer provided by LUMINEQ (Beneq Oy, Espoo, Finland). First, a 150 nm thick Al_2_O_3_ dielectric layer was grown by ALD. It was then followed by several ZnS (50 nm)/Y_2_O_3_:Eu (40 nm) multilayers. Finally, another 150 nm thick Al_2_O_3_ layer was deposited on the structure. The 1720 nm thick device was finalized by depositing a top contact. A schematic illustration of the device is presented in Figure 1. While it is possible to use a transparent top contact for a fully transparent device, for merely convenience purposes, top contact stripes of aluminum were sputtered here using a mechanical mask. The crossing of the ITO transparent contact and the aluminum stripes, which also comprises the sandwich multilayer Al_2_O_3_/ZnS/Y_2_O_3_:Eu/Al_2_O_3_ structure, creates a passive matrix with a pixel size of 3 × 5 mm^2^. Note that prior to the deposition of the top Al_2_O_3_ layer, the multilayer sequence was always completed with a ZnS top layer.

A SE400adv ellipsometer (SENTECH Instruments GmbH, Berlin, Germany) using a 633 nm wavelength at 70° angle of incidence, was used to determine the growth per cycle (GPC) for each material. GPC values were subsequently used to determine the thickness of the different layers. The crystallinity of Y_2_O_3_:Eu and ZnS thin films was investigated by X-ray diffraction (XRD) using the Cu Kα line in a Rigaku SmartLab (Rigaku Europe SE, Neu-Isenburg, Germany) high-resolution X-ray diffractometer equipped with in-plane arm. The XRD data were analyzed using the HighScore Plus 4.6 (PANalytical B.V., Almelo, The Netherlands). Photoluminescence (PL) emission was measured from Y_2_O_3_:Eu thin film phosphors with a Hitachi F-7100 Fluorescence Spectrophotometer (Hitachi High-Tech Analytical Science Ltd., Abingdon, UK) equipped with a 150 W xenon lamp. Measurements were performed at room temperature with an excitation slit of 5 nm, emission slit of 2.5 nm, and a photomultiplier tube voltage of 400 V. To determine the excitation wavelength, excitation spectra were recorded for maximum emission at 612 nm. Electroluminescent devices were powered by a Hewlett Packard 6811a source using AC mode at a frequency of 1 kHz. Electroluminescence spectra were recorded using a Konica Minolta CS-2000 spectrometer (Konica Minolta Sensing Europe B.V., Nieuwegein, The Netherlands) with a measurement angle of 1°.

For the calculation of the EL device efficiency, the Sawyer–Tower circuit was used to determine the charge density versus voltage (Q–V) characteristic. The used circuit is composed of a sense capacitor connected in series with the EL device. The total capacitance of the circuit was determined using a Fluke 76 digital multimeter. Data from the Q–V plot were acquired by measuring the voltage at each of the device terminals using a WaveSurfer 3104z (Teledyne Lecroy, Teledyne GmbH, Heidelberg, Germany) oscilloscope. The charge (Q) of the device could be determined by multiplying the output voltage by the total capacitance of the circuit [32]. Simulations were performed using LTspice XVII. 

## 3. Results

To optimize the emission of the Eu-doped Y_2_O_3_ thin film phosphors, films with three different Y_2_O_3_:Eu_2_O_3_ ratios were grown. Thus, three Eu doping concentrations (2:2, 3:2, and 4:2) were obtained by changing the number of Y_2_O_3_ and Eu_2_O_3_ sequences. As an example, a 4:2 doping configuration refers to a Y_2_O_3_:Eu thin film layer in which 4 layers of Y_2_O_3_ (Y(MeCp)_3_/N_2_/H_2_O/N_2_) were followed by 2 layers of Eu_2_O_3_ (Eu(Thd)_3_/N_2_/O_3_/N_2_/H_2_O/N_2_) during the ALD process_._ Taking into consideration Y_2_O_3_ and Eu_2_O_3_ densities, growth rates on Si substrate, and assuming that the Y_2_O_3_ and Eu_2_O_3_ films are stoichiometric, the 2:2, 3:2 and 4:2 doping configurations lead to calculated Eu concentrations of 16, 11, and 9 mol%, respectively.

### 3.1. Characterization of Y_2_O_3_:Eu and ZnS Thin Films 

**Figure 2:**
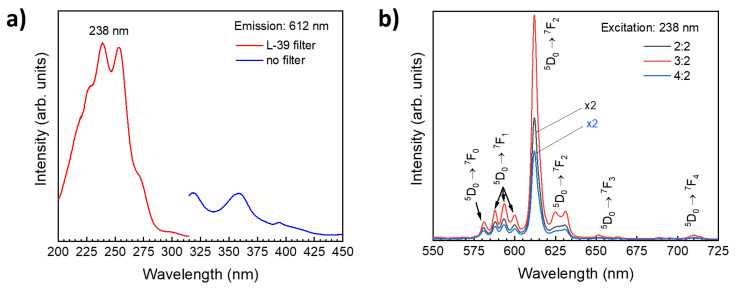
(**a**) Excitation spectra and (**b**) emission spectra of ALD Y_2_O_3_:Eu thin films prepared with different Eu concentrations. The measurements were performed at room temperature.

Figure 2a shows excitation spectra for a maximum emission at 612 nm, measured between 200 and 450 nm on a Y_2_O_3_:Eu sample grown on Si with a Y_2_O_3_:Eu_2_O_3_ layer ratio of 2:2. The excitation spectrum between 200 and 315 nm was measured using a Hitachi L-39 (UV-39) cutoff filter to remove a high intensity Rayleigh scattering peak located between 288 and 315 nm. The spectra show that the highest emission at 612 nm is obtained for an excitation of 238 nm. Figure 2b shows emission spectra for Y_2_O_3_:Eu_2_O_3_ layer ratios of 2:2, 3:2, and 4:2. The emission spectra were recorded for the excitation wavelength of 238 nm which was deduced from the excitation spectrum in Figure 2a. All PL spectra show an emission between 575 and 650 nm with a sharp line located at 612 nm. This red color emission is typical of the Eu^3+ 5^D_0_ → ^7^F_J_ (J = 0, 1, 2, 3, and 4) transitions. Note that Y_2_O_3_:Eu samples grown with 2:2 and 4:2 doping configurations show much lower emission intensities compared to the sample grown with a doping concentration of 3:2. 

**Figure 3:**
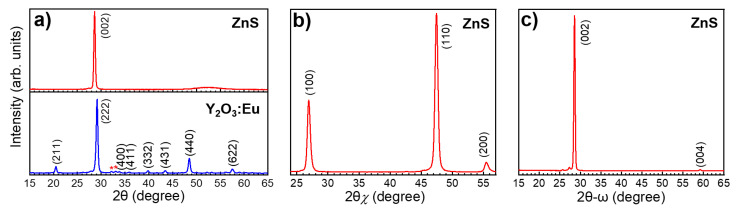
(**a**) Grazing incidence XRD for Y_2_O_3:_Eu and ZnS samples grown by ALD on Si substrates, (*) marks traces of monoclinic phase. The Y_2_O_3:_Eu sample was prepared with a 3:2 cycle ratio. (**b**) XRD spectrum for the ZnS sample measured in in-plane measurement mode. (**c**) XRD spectrum for the ZnS sample in the 2θ-ω measurement mode.

Figure 3a shows grazing incidence X-ray diffractograms for Y_2_O_3_:Eu and ZnS thin films measured between 15 and 65°. The Y_2_O_3_:Eu sample was prepared with a 3:2 (Y_2_O_3_:Eu_2_O_3_) cycle ratio. The Y_2_O_3_:Eu XRD diffractogram shows that the main phase of the film is polycrystalline (randomly orientated) cubic (pattern number 00-041-1105; Ia3) with some traces of monoclinic phase (marked with asterisk). The grazing incidence XRD data of the ZnS sample show clearly that the sample is highly orientated as only the (002) reflection is observed. The wide bump, between 45 and 60°, most likely originates from the substrate.

Further proof of the orientation was obtained by performing an in-plane measurement that probes the crystalline planes perpendicular to the surface normal as shown in Figure 3b. One can see only (hk0) family of planes meaning that (00l) planes are strongly orientated parallel to the surface. On Figure 3c, which shows the 2θ-ω measurement for the ZnS sample, the hump disappears supporting the idea that it was originated from the substrate. The peak at 59.1° reveals the (004) reflection related to the (002) intense reflection.

### 3.2. Red Electroluminescent Device

**Figure 4:**
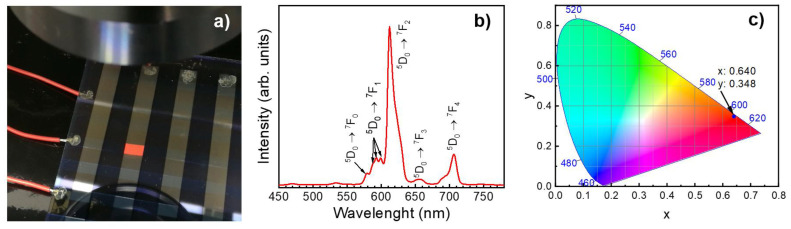
(**a**) Photograph of a 3 × 5 mm^2^ red Y_2_O_3_:Eu/ZnS-based EL pixel emitting an intensity of 40 cd/m^2^. The pixel was measured under normal room lighting. (**b**) Electroluminescence spectrum of the pixel shown in (**a**). (**c**) CIE 1931 chromatography diagram deduced from the spectrum in (**b**).

Figure 4a shows a photograph of a 3 × 5 mm^2^ red Y_2_O_3_:Eu/ZnS-based EL pixel under a sinusoidal excitation of 1 kHz measured at 280 Vrms. The photograph was taken with a digital camera in automatic mode under normal room lighting. For this pixel, a brightness of 40 cd/m^2^ was measured. Figure 4b shows the electroluminescence spectrum, at maximum luminance, of the Y_2_O_3_:Eu/ZnS EL device with a 3:2 (Y_2_O_3_:Eu_2_O_3_) cycle ratio. The EL spectrum, which was measured under an operating voltage of 280 Vmrs and a frequency of 1 kHz, clearly shows the typical ^5^D_0_ → ^7^F_J_ (J = 0, 1, 2, 3, and 4) transitions in Eu^3+^ emission centers. The sharp ^5^D_0_ → ^7^F_2_ line is located at 612 nm. Note the prominent ^5^D_0_ → ^7^F_4_ emission at 708 nm. The 1931 CIE color coordinates shown in Figure 4c were deduced from the EL spectrum in Figure 4b using OriginLab Chromaticity Diagram script (Origin Pro 2019, Northampton, MA, USA). Thus, the obtained red color emission corresponds to (x, y) values of (0.640, 0.348).

**Figure 5:**
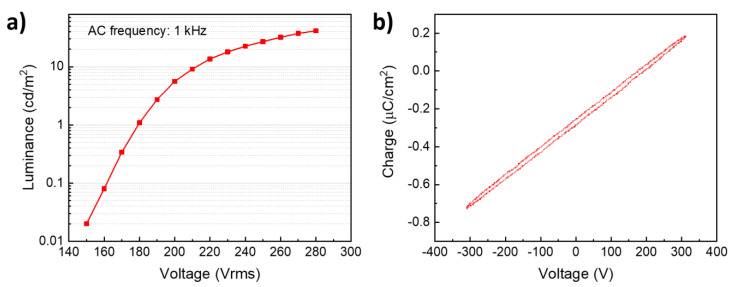
(**a**) Luminance versus the applied voltage and (**b**) charge–voltage (Q-V) characteristics for the Y_2_O_3_:Eu/ZnS electroluminescent device under a sinusoidal wave with a frequency of 1 kHz.

Figure 5a shows the luminance versus applied voltage characteristics of the Y_2_O_3_:Eu/ZnS electroluminescent device under a sinusoidal excitation of 1 kHz. The device shows a maximum brightness of 40 cd/m^2^ at 280 Vrms. The threshold voltage of the device is not well-defined; it can, however, be considered as the voltage needed for the generation of 1 cd/m^2^ [32]. Here, a luminance of 1 cd/m^2^ is achieved for an excitation voltage of 180 Vrms. Figure 5b shows Q–V characteristics of a ZnS/Y_2_O_3_:Eu EL device, measured at 40 Vrms above the threshold voltage and 1 kHz sinusoidal wave. The measured sense capacitor and total capacitance of the circuit were 171 nF and 6.24 nF, respectively. The input power density, which was calculated by multiplying the area of the graphic in Figure 5b to the applied frequency, was determined to be 153 W/m^2^. Based on these values, an efficiency of 0.28 Lm/W was calculated. Note the Y axis of the Q–V curve which is not centered in the position (0, 0) coordinates of the graphic.

## 4. Discussion

Y_2_O_3:_Eu, ZnS, and Al_2_O_3_ thin films were successfully grown by ALD at 300 °C using commercial precursors. The processing temperature was limited to 300 °C because of the decomposition temperature of the metalorganic precursors and O_3_. Y_2_O_3:_Eu thin film samples, grown with different Eu concentrations, show clearly red emission with a maximum intensity at 612 nm. This line is related to the ^5^D_0_ → ^7^F_1_ magnetic dipole transition of Eu^3+^ [34]. With the process conditions described in this work, the optimum Eu concentration was found to be about 11 mol%. While a lower Eu concentration of 9 mol% led to lower PL intensities as expected, the well-known quenching that arises from energy transfer between the Eu^3+^ luminescent centers was observed for a Eu concentration of 16 mol%. These values are close to the ones reported by H. Huang et al. [35] in comparison with the optimum Eu concentration values of 20 and 5 mol% reported by J. Kaszewski et al. [25] and Y. Kumar et al. [27], respectively.

In a classic DSD electroluminescent device structure, an ideal phosphor should have a polycrystalline structure [32]. Therefore, the polycrystalline nature of our ALD Y_2_O_3:_Eu and ZnS thin film layers is advantageous to the multilayer Y_2_O_3_:Eu/ZnS electroluminescent device. Furthermore, in comparison with other reported Y_2_O_3_:Eu electroluminescent devices [36,37,38], our low processing temperature of 300 °C offers the possibility of building devices on some temperature-resistant polymer flexible substrates [39]. 

An all-in-one growth step for the Al_2_O_3_ dielectric, ZnS, and Y_2_O_3_:Eu phosphor layers was used for the fabrication of our EL device by ALD. In contrast to the photoluminescence spectrum, the electroluminescence spectrum shows a prominent ^5^D_0_ → ^7^F_4_ emission at 708 nm. This could be due to the lower sensitivity of the PL equipment in comparison with the EL equipment, since most photomultiplier tubes have lower sensitivity in the ^5^D_0_ → ^7^F_4_ transition region [34]. At 280 Vrms and under a sinusoidal excitation of 1 kHz, with the growth conditions reported in this paper, we achieved high-purity red color emission with an intensity up to 40 cd/m^2^. This intensity could be significantly increased by further optimization of the different device layers, i.e., optimization of Y_2_O_3_:Eu and ZnS thicknesses and the dielectric layer (here a mere Al_2_O_3_ layer was used). Using multilayer structures, red and green Y_2_O_3_/Y_2_O_2_S-based electroluminescent devices with luminance up to 137 cd/m^2^ (at 150 Vrms) and 124 cd/m^2^ (at 300 Vrms), respectively, were reported by T. Suyama et al. [19] and K. Ohmi et al. [20]. While those values are higher than the ones we obtained for our devices, devices in [19,20] were measured under an excitation frequency of 5 kHz. Frequency has been reported to significantly influence the electroluminescence emission intensity. As an example, luminance values could be increased from 15 to 350 cd/m^2^ in CaYS:Eu electroluminescent devices by increasing the frequency from 50 Hz to 1 kHz [7]. 

**Figure 6:**
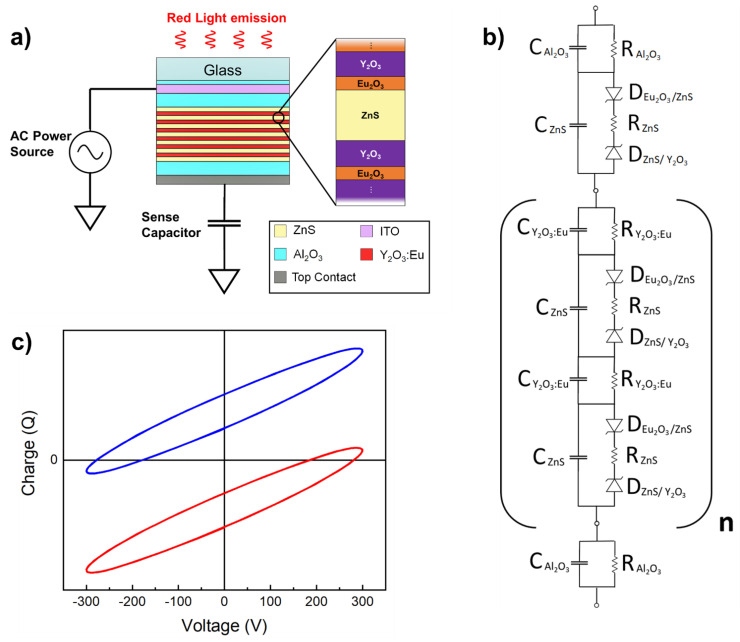
(**a**) Upside down representation of the 2D schematic of the Y_2_O_3_:Eu/ZnS EL device connected in a Sawyer-Tower circuit schematic with an amplification scheme of the ZnS layer and its surroundings. (**b**) Equivalent circuit of the Y_2_O_3_:Eu/ZnS EL device. (**c**) Simulated Q-V characteristics when (red) ITO is connected to the power supply and the top contact is connected to the sense capacitor; and (blue) ITO is connected to the sense capacitor and the top contact is connected to the power supply.

While it is difficult to compare the efficiency of our device with other red electroluminescent devices due to different measurement conditions, the calculated efficiency of 0.28 Lm/W for our ZnS/Y_2_O_3_:Eu multilayer EL device is lower than the 0.8 Lm/W value reported for the ZnS:Mn EL device with red filter and measured with a frequency of 60 Hz [16]. Q–V characteristics usually appear in a trapezoid shape where physical quantities such as threshold voltage, threshold voltage of the phosphor layer, threshold charge density, and transferred charge density are well-defined [32]. The elliptic shape of our Q–V characteristics is due to the multilayer structure of the ZnS/Y_2_O_3_:Eu EL device and possible presence of leakage current in the phosphor layer. Our Q–V curve appears negatively biased when the ITO layer of the EL device is connected to the power supply and the top contact is connected to the sense capacitor in the Sawyer–Tower circuit, as shown in Figure 6a. However, when the connections are inverted (the top contact is connected to the power supply and the ITO layer is connected to the sense capacitor), the Q–V curve appears positively biased. Therefore, one possible explanation for this behavior is the asymmetric structure of the device. During the growth process, each phosphor layer starts with the deposition of Y_2_O_3_ and finishes with Eu_2_O_3_ making ZnS surrounded on one side by Y_2_O_3_ and on the other by Eu_2_O_3_ as shown in Figure 6a. We believe this asymmetry might favor charge accumulation. 

The Q–V characteristics could be reproduced by simulating the equivalent circuit (Figure 6b) of the EL device in the Sawyer–Tower circuit. Figure 6c shows the simulation results of two different scenarios: (i) in red, where the Sawyer–Tower circuit has the EL device with the ITO layer connected to the power supply and the top contact connected to the sense capacitor, as depicted in Figure 6a; and (ii) in blue, where the data were simulated with the top contact connected to the power supply and the ITO layer to the sense capacitor. This simulation requires high voltages and one Zener diode (related to the ZnS/Y_2_O_3_ or Eu_2_O_3_/ZnS interfaces) with higher threshold voltage than its counterpart. The simulation in Figure 6c matches Figure 5b when the Zener diode D_ZnS/Y2O3_, which is related to the ZnS/Y_2_O_3_ interface, has a larger breakdown voltage than D_Eu2O3/ZnS_.

## 5. Conclusions

In this work, we demonstrate the feasibility of transparent red Y_2_O_3:_Eu-based electroluminescent devices by atomic layer deposition at relatively low temperature. Y_2_O_3:_Eu, ZnS, and Al_2_O_3_ thin films and related multilayer structure devices were prepared at 300 °C. XRD measurements showed high crystallinity of the Y_2_O_3:_Eu and ZnS films. Photoluminescence and electroluminescence measurements showed a bright red emission of the phosphors and electroluminescent devices, respectively. A luminance up to 40 cd/m^2^ and an efficiency of 0.28 Lm/W were achieved. Further optimization of the phosphor and EL device is expected to lead to higher emission intensities.

## Figures and Tables

**Table 1:** Pulsing sequences and corresponding pulse time and purge time for the thin films prepared by atomic layer deposition (ALD). Growth per cycle (GPC) values displayed on the table were deduced from ellipsometry measurements.

Process	Pulsing Sequence	Pulsing Time (s)	GPC [nm]
Y_2_O_3_	Y(MeCp)_3_/N_2_/H_2_O/N_2_	2/6/0.2/7	0.16
Eu_2_O_3_	Eu(Thd)_3_/N_2_/O_3_/N_2_/H_2_O/N_2_	3/7/5/7/0.2/7	0.03
Al_2_O_3_	AlMe_3_/N_2_/H_2_O/N_2_	0.5/5/0.3/5	0.10
ZnS	Zn(OAc)_2_/N_2_/H_2_S/N_2_	2.5/6/0.3/3	0.24

## Data Availability

Data sharing is not applicable to this article.
